# Detection of Sugar Chain Expression in Hydatidiform Mole Using Lectin Histochemistry

**DOI:** 10.5812/ircmj.3997

**Published:** 2013-05-05

**Authors:** Fatemeh Atabaki Pasdar, Alireza Khooei, Alireza Fazel, Mahmoud Mahmoudi, Fatemeh Tavassoli

**Affiliations:** 1 Department of Anatomy & Cell Biology, Mashhad University of Medical Sciences, Mashhad, IR Iran; 2 Department of Pathology, Imam Reza Hospital, Mashhad University of Medical Sciences, Mashhad, IR Iran; 3 Immunology Research Center, Bu-Ali Research Institute, Mashhad University of Medical Sciences, Mashhad, IR Iran; 4 Department of Obstetrics & Gynecology, Imam Reza Hospital, Mashhad University of Medical Sciences, Mashhad, IR Iran

**Keywords:** Hydatidiform Mole, Lectins, Histochemistry, Carbohydrates

## Abstract

**Background:**

Hydatidiform moles carry a significant risk for developing persistent gestational trophoblastic disease. Lectins are useful tools to identify cellular glycosylation pattern and changes in glycosylation that occur during growth, development, differentiation and also, during disease states.

**Objectives:**

Considering the changes in glycosylation that occur during cell proliferation, differentiation and transformation, the aim of the present study was to evaluate the sugar chain expression in hydatidiform mole by using HRP-conjugated lectins.

**Materials and Methods:**

Lectin histochemistry with a panel of HRP-conjugated lectins comprising SBA, PNA, VVA, UEA-1, LTA, GS-І (B4) and WGA were performed in 20 molar (partial & complete moles) formalin-fixed, paraffin-embedded tissue samples.

**Results:**

The partial and complete moles generally showed similar reactivity with all used lectins. None of lectins reacted with villous cytotrophoblasts, whereas 4 of 7 lectins comprising WGA, LTA, UEA-І and PNA (after pretreatment with neuraminidase) showed a moderate to strong reactivity with villous syncytiotrophoblasts in both partial and complete hydatidiform moles. The villous stroma reacted with all used lectins except VVA.

**Conclusions:**

Our histochemical findings showed a relatively heavy glycosylation of syncytiotrophoblasts of both partial and complete molar tissues, which was prominent in apical portion. This may play a role in their capacity to increase trophoblastic proliferation.

## 1. Background

Gestational Trophoblastic Disease is a group of interrelated tumors originating from the placenta. Hydatidiform Mole is the most common form, which is abnormal pregnancy characterized by hydropic swelling of placental villi and trophoblastic hyperplasia; this includes Partial and Complete Hydatidiform Mole (1). Hydatidiform moles carry a significant risk for developing persistent Gestational Trophoblastic Disease, with the higher incidence in patients with Complete Hydatidiform Mole (10%-30%) than patients with Partial Hydatidiform Mole (0.5%-5%) (2). Hydatidiform moles occur in approximately 1 in every 1500 pregnancies in Europe and North America .This is 3-10 times higher in some countries of Latin America, the Middle East, and the Far East (3, 4). These studies demonstrated that women of Asian origin are at a higher risk of developing moles than others. The importance of cell surface carbohydrates is particularly evident from the finding of their variation in expression during embryonic development and cell differentiation. Numerous data has been accumulated showing that malignant transformation is also associated with various alternations in the expression of cell surface sugar chains that might indicate that carbohydrates play a role in malignant transformation (5-7). Lectins are proteins or glycoproteins which have a specific binding affinity for the carbohydrate structures on glycoconjugates of cells and tissues. This allows investigators to identify cellular glycosylation patterns and changes in glycosylation that occur during growth, development, differentiation, and changes that occur during disease states (8).

## 2. Objectives

The aim of present study was to evaluate the sugar chain expression in hydatidiform mole by using HRP-conjugated lectins.

## 3. Materials and Methods

### 3.1. Case Selection

Formalin-fixed, paraffin-embedded molar tissue samples of some patients diagnosed in Departments of Pathology of Imam Reza and Qhaem, two teaching hospitals of Mashhad University of Medical Sciences were gathered. Tissue specimens consisted of 10 complete and 10 partial hydatidiform moles. Gestational age ranged from 8 to 16 weeks (mean, 11.6 weeks). Tissue sections of the specimens were stained with routine hematoxylin-eosin and histopathologically reviewed for confirmation of diagnosis and selection for the best region for lectin histochemistry.

### 3.2. Lectin Histochemistry

4-5 micrometer tissue sections were deparaffinized in xylene and rehydrated through graded dilutions of ethanol. Endogenous peroxidase activity was blocked by preincubation of tissues with 0.5% hydrogen peroxide in methanol for 15 min at room temperature, and then washed in PBS. Tissue sections then were covered by HRP-conjugated lectins ( UEA-1, LTA, PNA, SBA , GS-І(B4), VVA and WGA), which were purchased from Sigma-Aldrich company and diluted in 0.1 M PBS to reach the final concentration 10μg of lectins, and placed in a humid chamber for 2 hours at room temperature. The tested lectins and their major sugar specificities are listed in Table 1. After incubation, excess unbound reagent was removed by washing 3 times in PBS and the reaction was then developed in 0.03% diaminobenzidine in PBS with 0.006 % hydrogen proxidase and after 10 min, reaction was stopped by washing in tap water. The slides were counterstained with alcian-blue 1%, dehydrated and mounted in synthetic resin ( 9 - 11 ). Negative control samples were made by the same procedure without lectins and known positive tissues for each lectin were used as positive controls. According to previous studies, in some experiments sialic acid was removed by pretreating the sections for 18h at 37 ºC in sodium acetate buffer 0.25 M pH 5.5, containing 0.1 unit/ml neuraminidase, prior to application of PNA lectin ( 12 , 13 ). All the slides were stained in the same batch to eliminate interbatch variation. Reactivity to these lectins was assessed in villous trophoblasts and core stroma. Ten fields examined for each section using a light microscope, magnification × 200. The intensity of staining were graded subjectively by two observers and scored as – (negative), + (weak), ++ (moderate) and +++ (strong).

**Table 1.** UsedLectins and Their Major Specificities (13,14)

Name of Lectin	Abbreviation	Major Sugar Specificity
**Soybean Agglutinin**	SBA	α/β-D-GalNAc^a^ > D-Gal
**Peanut Agglutinin**	PNA	D-Gal^a^ (β1-3)-D-GalNAc^a^
**Vicia** **Villosa**	VVA	GalNAc
**Ulex** **Europaeus** ** Agglutinin І**	UEA І	α -L-fuc^a^
**Lotus ** **Tetragonolobus**	LTA	α -L-fuc
**Griffonia** **simplicifolia**	GS-І ( B4)	α–Gal
**Wheat Germ Agglutinin**	WGA	(GlcNAc)n , Sialic Acid

^a^Abbreviations: GalNAc, N-acetylgalactosamine; Gal, galactose; fuc, fucose; GlcNAc; N-acetylglucosamine

## 4. Results

The partial and complete moles generally showed similar reactivity with all used lectins. The results were summarized in Table 2.

### 4.1. SBA

This lectin did not react with syncytiotrophoblasts and cytotrophoblasts, but moderately reacted with villous stroma.

### 4.2. PNA

**Figure 1.**
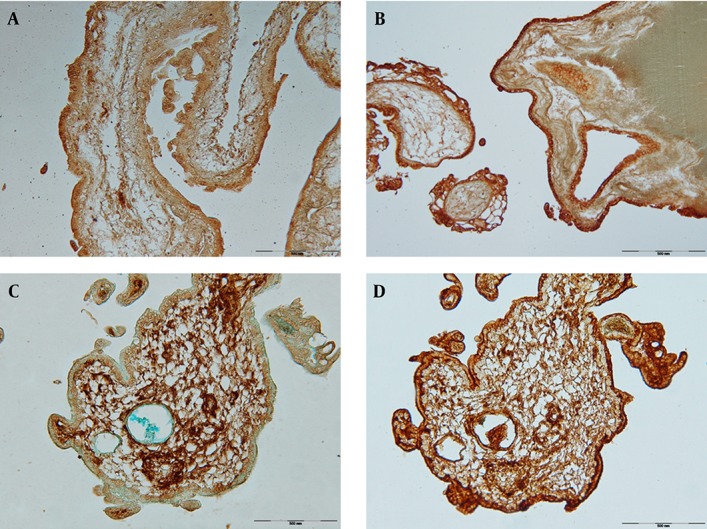
UEA І Reactivity in Complete Hydatidiform Mole A: WGA reactivity in partial hydatidiform mole B: PNA reactivity before neuraminidase digestion C: and after neuraminidase digestion D: in partial hydatidiform moles, Scale bar = 500µm

No reaction was observed in syncytiotrophoblasts and cytotrophoblasts. The villous stroma showed moderate reactivity (Figure 1 c), after neuraminidase treatment moderate reactivity was observed in syncytiotrophoblasts, which was most pronounced in apical portion .The reactivity of other components of villi did not altered (Figure 1 d).

### 4.3. VVA

The various components of the placental villi did not react with this lectin.

### 4.4. UEA І

The syncytiotrophoblasts showed moderate reactivity. No reactivity was observed in cytotrophoblasts. The villous stroma moderately reacted with this lectin (Figure 1 a).

### 4.5. LTA

A moderate reactivity was observed in syncytiotrophoblasts but the cytotrophoblasts did not react. The villous stroma showed a moderate reactivity.

### 4.6. GS-І (B4)

The syncytiotrophoblasts and the cytotrophoblasts did not react with this lectin. A moderate reaction was observed in villous stroma.

### 4.7. WGA

The syncytiotrophoblasts showed a strong reactivity, which was most pronounced in apical portions. The cytotrophoblasts did not react with this lectin. A weak reaction was observed in stromal cells (Figure 1 b).

**Table 2.** Lectin Binding Pattern in Hydatidiform Mole

Lectins	Cell Populations		
	Syncytiotrophoblast	Cytotrophoblast	Villous Stroma
**SBA**	−^a^	−	++
**PNA**	−	−	++
**PNA-N**	++^b^	−	++
**VVA**	−		−
**UEA- І**	++	−	++
**LTA**	++	−	++
**GS-І (B4)**	−	−	++
**WGA**	+++^c^	−	+^d^

^a^−: negative

^b^++: moderate

^c^+++: strong

^d^+: weak

## 5. Discussion

Lectins have been found to serve as markers of proliferation, differentiation and malignant transformation. Lectin binding studies can be performed on fixed, paraffin-embedded tissues, including archival tissues that have been stored for prolonged periods. There have been several studies on the lectin-binding properties of chorionic villi in normal pregnancies, (14-17) but we found a few studies about molar pregnancies. Our results indicate that HRP-conjugated lectins used in this study react differentially with various components of molar tissue; however these reactions were generally similar in partial and complete hydatidiform moles. None of lectins used in this study reacted with cytotrophoblasts, whereas 4 of 6 lectins reacted with syncytiotrophoblasts which was prominent in apical portion. Cytotrophoblast is the trophoblastic stem cell, whereas syncytiotrophoblast is the terminally differentiated cell that produces most of the placental hormones (18). The apical portion of the trophoblast corresponds to the microvillus brush border that has been shown, in previous studies, to be heavily glycosylated (19, 20). The brush border of the syncytiotrophoblast layer of the placenta forms the first barrier separating the maternal blood from the fetal circulation and is important in the exchange of nutrients, hormones and waste products between the mother and the fetus.

The strong reactivity of syncytiotrophoblasts with WGA which was most pronounced in apical portions could be due to increased N-acetylglucosamine and/or sialic acid, however Tatsuzuki et al. demonstrated that the brush border of syncytiotrophoblast layer of human term placenta strongly expressed GlcNAc and weakly expressed sialic acid (14). This is consistent with previous study performed by Juane et al., they showed that this increased reactivity was correlated with growth and proliferation of trophoblasts in trophoblastic disease (12) in contrast, Thrower et al. showed a weak reactivity in syncytiotrophoblast of all examined specimens comprising normal term pregnancy, ectopic pregnancy and molar pregnancies, using WGA lectins (17). Proteolytic treatments of paraffin sections performed in this study affect lectin histochemistry, (21) on the other hand, different detection methods may affect the results of lectin histochemistry, and they used biotinylated lectins. As described in previous studies, (14, 22). PNA did not bind with villous syncytiotrophoblast and cytotrophoblast prior to neuraminidase treatment. However after pretreatment with neuraminidase, the villous syncytiotrophoblasts showed moderate binding with PNA in partial and complete moles, this concurs with previous studies (12, 13, 16) PNA lectin has been shown to have specificity for D-Gal (1-3)-D-GalNAc which is supposed to be the antigenic determinant for the Thomsen-Friedenreich antigen or TF-Ag (17, 20). This antigen is normally present in many structures, Ritcher et al reported expression of this antigen on trophoblastic cells, (23) but is considered cryptic, because it is usually covered by a terminal sialic acid. Pretreatment of tissue section with neuraminidase prior to application of PNA lectins would expose this T-Ag as was shown in the normal placenta (12). In the present study, for the detection of fucosyl residue two different types of lectins, UEA І and LTA, were employed, reactivity with LTA suggests the presence of reactive sites containing α-L-Fucose which bind via α (1-6) linkage to penultimate glucosaminyl residues and/or difucosylated oligosaccharides, (24) while reactivity with UEA І, indicates the presence of α-L-Fucose bound via β1,2 linkage to penultimate D-galactose-(β1-4)-N-acetyl-D-glucosamine residues (25). In both partial and complete moles, LTA and UEA І, reacted moderately with Syncytiotrophoblast which was prominent in apical portion, thus revealing the presence of α-L-Fucose with both types of linkage, Sgambatti et al. reported the reaction with LTA and UEA І lectins observed in apical portion of syncytiotrophoblasts of normal placenta which increased during the late stage of placentation, whereas no binding of these lectins was seen in trophoblasts of human placenta of pregnancies complicated by intrauterine growth retardation, and this suggests the role of α-L-fucose in nutrient transfer (13). In this study, the moderate reactivity of Syncytiotrophoblasts with LTA and UEA І, may be due to increased growth and proliferation of trophoblast in molar pregnancies which demand more exchange of nutrients and metabolic products. Our histochemical findings showed a relatively heavy glycosylation of syncytiotrophoblasts of both partial and complete molar tissues, which was prominent in apical portion which may play a role in their capacity to increased trophoblastic proliferation.
